# Vgsc-interacting proteins are genetically associated with pyrethroid resistance in *Aedes aegypti*

**DOI:** 10.1371/journal.pone.0211497

**Published:** 2019-01-29

**Authors:** Corey L. Campbell, Karla Saavedra-Rodriguez, Tristan D. Kubik, Audrey Lenhart, Saul Lozano-Fuentes, William C. Black

**Affiliations:** 1 Department of Microbiology, Immunology and Pathology, Colorado State University, Fort Collins, Colorado, United States of America; 2 Division of Parasitic Diseases and Malaria, Center for Global Health, Centers for Disease Control and Prevention, Atlanta, Georgia, United States of America; Centro de Pesquisas René Rachou, BRAZIL

## Abstract

Association mapping of factors that condition pyrethroid resistance in *Aedes aegypti* has consistently identified genes in multiple functional groups. Toward better understanding of the mechanisms involved, we examined high throughput sequencing data (HTS) from two *Aedes aegypti aegypti* collections from Merida, Yucatan, Mexico treated with either permethrin or deltamethrin. Exome capture enrichment for coding regions and the AaegL5 annotation were used to identify genes statistically associated with resistance. The frequencies of single nucleotide polymorphisms (SNPs) were compared between resistant and susceptible mosquito pools using a contingency χ^2^ analysis. The -log_10_(χ^2^ *p* value) was calculated at each SNP site, with a weighted average determined from all sites in each gene. Genes with -log_10_(χ^2^ *p* value) ≥ 4.0 and present among all 3 treatment groups were subjected to gene set enrichment analysis (GSEA). We found that several functional groups were enriched compared to all coding genes. These categories were transport, signal transduction and metabolism, in order from highest to lowest statistical significance. Strikingly, 21 genes with demonstrated association to synaptic function were identified. In the high association group (n = 1,053 genes), several genes were identified that also genetically or physically interact with the voltage-gated sodium channel (*VGSC*). These genes were eg., *CHARLATAN (CHL)*, a transcriptional regulator, several ankyrin-domain proteins, *PUMILIO* (*PUM*), a translational repressor, and *NEDD4* (E3 ubiquitin-protein ligase). There were 13 genes that ranked among the top 10%: these included *VGSC*; *CINGULIN*, a predicted neuronal gap junction protein, and the aedine ortholog of *NERVY (NVY)*, a transcriptional regulator. Silencing of *CHL* and *NVY* followed by standard permethrin bottle bioassays validated their association with permethrin resistance. Importantly, *VGSC* levels were also reduced about 50% in *chl-* or *nvy-*dsRNA treated mosquitoes. These results are consistent with the contribution of a variety of neuronal pathways to pyrethroid resistance in *Ae*. *aegypti*.

## Introduction

The major arbovirus vector *Aedes aegypti* continues to pose significant threats to human health in tropical and subtropical urban areas [1,2]. In southern Mexico, where dengue is hyperendemic [3], insecticides used for *Ae*. *aegypti* control include organophosphates for larval control and pyrethroids for adults [4]. The voltage-gated sodium channel (Vgsc, aaeN_AV_, LOC5567355) is one target of pyrethroid toxicity [5]. Increased resistance in *Ae*. *aegypti* to pyrethroid insecticides has been documented in many locations [6–9], and is associated with the presence of specific *VGSC* alleles. In addition, multiple lines of evidence indicate that metabolic resistance mechanisms may be equally important as target-site resistance [10–13]. Metabolic resistance to pyrethroids is generally described as a number of Vgsc-independent mechanisms derived from gene duplication, transcript overexpression, sequestration or increased reduction/oxidation (redox) activity. Cytochromes P450 (*CYP*), episilon class glutathione S-transferases (eGST) and esterases (EST) are major contributors to metabolic resistance, which are stimulated in response to oxidative stress [14–16].

To further increase our understanding of pyrethroid resistance mechanisms, the aim of the present study was to use association mapping to characterize allele frequency differences in genes for two *Ae*. *aegypti aegypti* field locations. One was located in the community of Viva Caucel in the city of Merida in the Mexican state of Yucatán and the other in Vergel in the same city. These locations were chosen because the association between *VGSC* genotype and permethrin knockdown resistance (*kdr*) phenotype were known for each site [9]. Specifically, Vgsc target site resistance loci V1016I and F1534C (house fly protein annotation), were present in each population. According to Vera-Maloof et al. 2015, in the Viva Caucel collection, the V1016I and F1534C mutations were present in 75% and 93% of individuals assayed, respectively. Similarly, in the Vergel collection, V1016I and F1534C were present in 80% and 98% of individuals assayed, respectively. Thus, in both locations, the F1534C allele was close to fixation. These characteristics indicated that the natural populations from which the collections were derived had already been selected for permethrin resistance.

Our hypothesis was that pyrethroid resistance-associated genes could be those with 1) chromosomal physical proximity to *VGSC*, resulting from a selective sweep, 2) selection due to direct activity in resistance or 3) genetic drift. For the present study, exome capture genomic DNA (gDNA) high throughput sequencing (HTS) data from both locations with two pyrethroid insecticides were analyzed to identify SNPs that differed significantly in frequency between susceptible and resistant pools in a given collection. We then highlighted those genes with significant genetic association to pyrethroid resistance. Genetic association refers to the concurrent presence of genotypic polymorphisms, as indicated by–log_10_(*p* value) scores from χ^2^ contingency table calculations, and the pyrethroid resistance phenotype. We used orthology information to assign functional attributes for all coding genes and used that information to identify those with statistical association to pyrethroid resistance. In addition, the gene set common to all treatment groups was subjected to GSEA [17]. Identification of genes common to both permethrin- and deltamethrin-resistant mosquitoes would give insight into overall pyrethroid resistance mechanisms. The identification of common mechanisms would also build support for increasing variability of rotating pyrethroid spraying regimens to reduce selection of resistant loci [18].

## Results and discussion

### Treatment and high throughput sequencing

**Fig 1:**
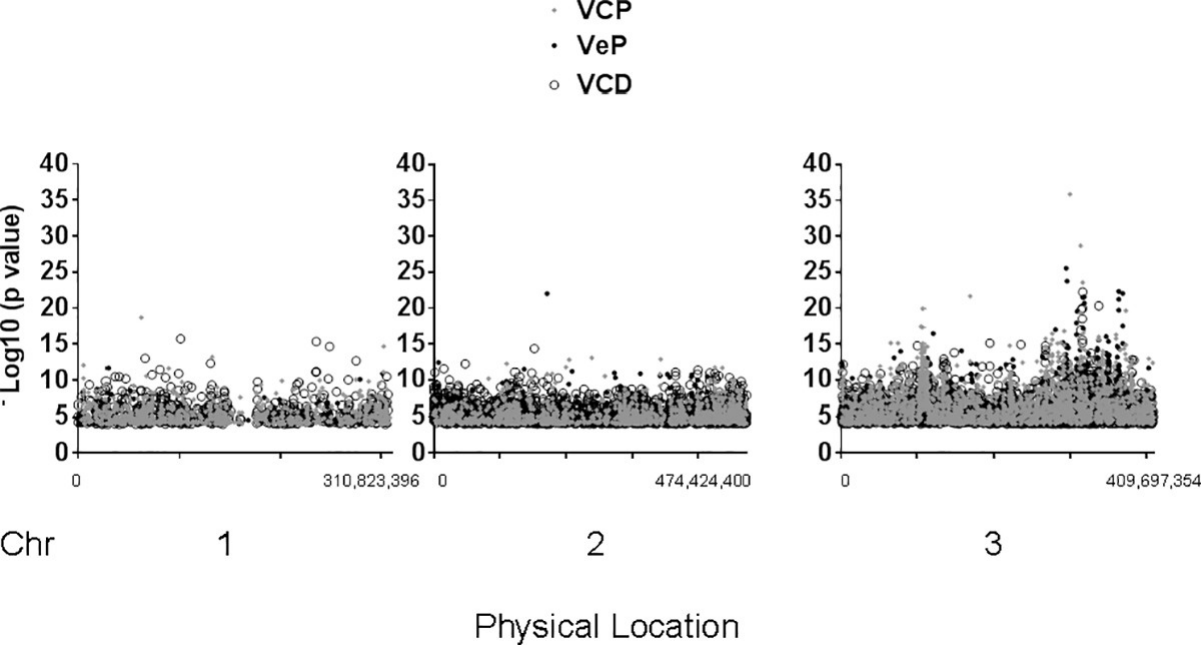
Genes with statistical association to pyrethroid resistance. Genes (FDR, p<0.01, -log_10_ (p value) of χ^2^ contingency calculation ≥ 4.0) in each of three collections, Viva Caucel permethrin (VCP, gray circle), Viva Caucel deltamethrin (VCD, open circle), and Vergel permethrin (VeP, black circle) were plotted according to the average physical position of each gene (AaegL5 genome build). X-axis shows beginning and end locations of polymorphisms on each chromosome.

A total of 3 pyrethroid treatment groups were evaluated for this work. Two groups, from the Viva Caucel location, were treated with either permethrin or deltamethrin. The third group, from the Vergel location, was treated with permethrin. Our rationale for evaluating combined data for type 1 and type 2 pyrethroids was 1) to identify genomic loci that are common among both treatment types and 2) reduce the identification of population-specific loci. Previously published bottle assay results are summarized in Table 1 [8,9]. Dual independent replicate pools of adult females (n = 25 per pool) from each population were prepared for a total of 12 library sets, comprising four from each location/treatment group (see Fig 1 legend). Then they were exome-capture enriched for genic regions [19,20]. From 27.25–86 million trimmed paired end reads were obtained for each replicate library (Table 2, S1 Table), and 94 to 98% of all trimmed reads aligned to the AaegL5 chromosome length reference sequence [21], which allowed for mapping of genes to their approximate physical location and eliminated the problems with unmapped genes that we experienced in previous studies [19].

**Table 1:** Bottle assays.

Collection-Treatment*	BioAssay	Total
Viva Caucel-Permethrin	Active at 1 hpt	94
	Knocked down at 4 hpt	95
Viva Caucel-Deltamethrin	Active at 1 hpt	111
	Knocked down at 4 hpt	92
Vergel-Permethrin	Active at 1 hpt	95
	Knocked down at 4 hpt	96

hpt, hours post-treatment; See also [8,9].

**Table 2:** Polymorphisms and coverage.

SNPs and Coverage	Viva Caucel—Permethrin		Viva Caucel—Deltamethrin		Vergel—Permethrin	
						
Monomorphic SNPs -Excluded	27,224,844		32,176,056		24,638,965	
Number of variant sites	31,358,228		36,447,261		29,417,399	
Coverage per nucleotide**-	-Min	61		60		62
	-Max	3469		3575		2893
	-Mean	367		276		329
	-Median	306		207		298

**, total coverage across all replicates

At each SNP, χ^2^ contingency table calculations determined statistical association of a given SNP to pyrethroid resistance. Resistance associated gene-wise scores were calculated from the weighted average–log_10_(*p* value) of the top 5% of SNP scores per gene (see Methods). Prior to further analysis, a multiple-testing adjustment (false discovery rate, FDR, alpha = 0.01) [22] and -log_10_(χ^2^ *p* value) ≥ 4.0 cut-offs were applied. Collection-specific details are shown in S2 Table. Gene-wise association scores above the cut-off were mapped relative to each gene’s physical position for each collection (Fig 1).

### Groups with high association to pyrethroid resistance

**Fig 2:**
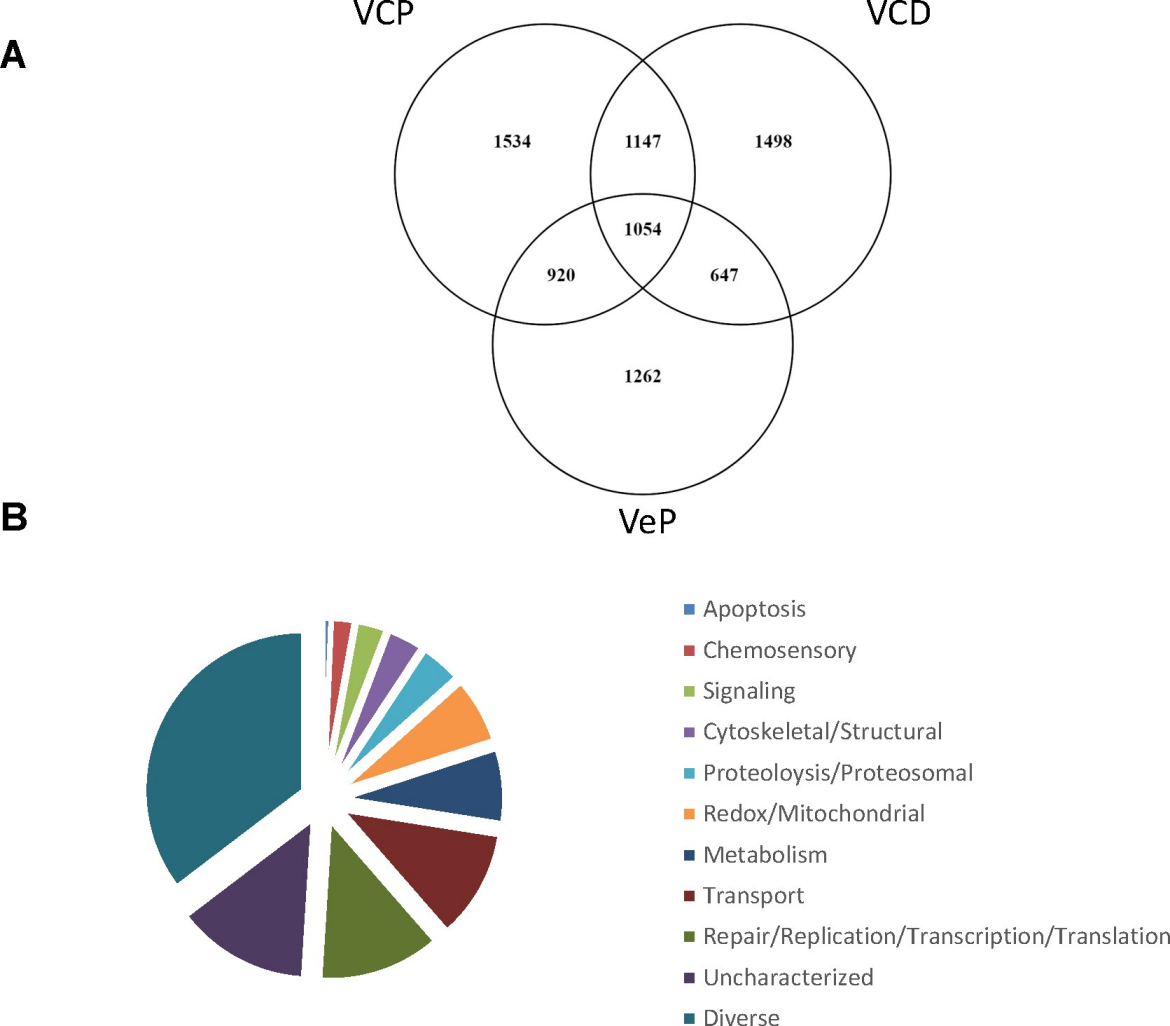
Genes with statistical association to resistance were common among all three collections (n = 1054). A) Venn diagram indicates common genes among all three treatment groups; Viva Caucel permethrin (VCP), Viva Caucel deltamethrin (VCD), and Vergel permethrin (VeP). B) Functional categories for genes in the high association set are listed from least abundant (top) to most abundant in the order of the diagram slices. X-axis shows beginning and end locations of polymorphisms on each chromosome.

**Fig 3:**
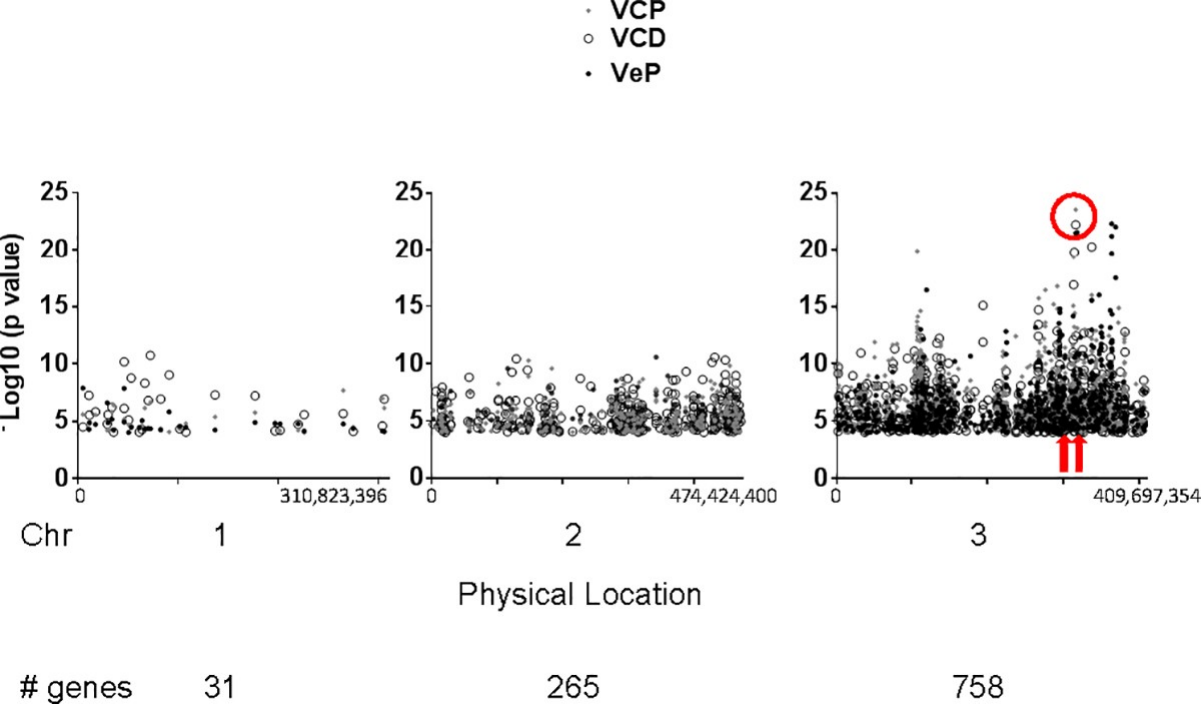
Physical positions of genes in high association subset (n = 1054). Genes were plotted according to their physical positions (AaegL5 reference). Y axis indicates weighted gene-wise average–log_10_(*p* value) of χ^2^ contingency table calculations. Red circle indicates approximate chromosomal location of the permethrin target site *VGSC* (nt positions 315,926,360–316,405,639) in VCP, VCD and VeP; red arrows show approximate location of *NVY* (nt 301,564,432–301,941,839) and *CHL* (nt 312,986,638–313,140,457).

To identify high association genes and functional categories associated with pyrethroid resistance among all three treatment groups, the intersection of genes above the thresholds were determined (Fig 2A, n = 1,054). One of these was a non-coding RNA and therefore was excluded from further analysis, leaving 1053 coding genes (S2 Table). This high association set was annotated by predicted function based on orthology to model organisms [23]. Overall functional groups for these genes are displayed in Fig 2B. Within the common gene set, the relative physical position of each gene and the corresponding genetic association values by collection are shown in Fig 3. Strikingly, though the common gene subset spanned all three chromosomes (S2 Table), most genes (1,023 of 1,053) were on chromosomes 2 and 3.

#### *VGSC* and associated genes

As expected, our tests revealed high statistical association of *VGSC*, the target of permethrin, in all three treatment groups (S2 Table). *VGSC* is located on the q arm of chromosome 3 [21,24] (Fig 3). Intriguingly, some genes in the high association group interact with *VGSC* in other organisms. For example, *CHL* (LOC5578279), a neuronal-specific zinc-Cys2His2 type transcriptional regulator [25], genetically interacts with the drosophilid ortholog of *VGSC* (*PARA*) [26]. Synaptic control of motoneuron function has been characterized in dipteran models of epilepsy, wherein increased frequency and regularity of calcium signaling waves, synaptic excitation, are indicative of pathology [26–29]. For example, in a drosophilid epilepsy model, reduced expression of *CHL* further increased sensitivity of the *PARA* bang-sensitive (*PARA* ^*bss*^) allele, which has a high basal sensitivity to paralysis. *PARA* ^*bss*^ was also sensitive to alterations in calcium levels [29], which is consistent with a functional interaction between Vgsc and calcium channels. Similarly, deltamethrin has been shown to reduce calcium channel efflux in mammalian and mosquito cell culture [30–32]. Consistent with this evidence, a calcium channel alpha subunit (*caα1*, LOC5564339) was also within the high association group.

The ankyrin protein domain is present in a large variety of structural proteins that link transmembrane proteins to the actin cytoskeleton [33,34]; they serve a variety of functions, including cytoskeletal anchoring and mechano-transduction of sensory responses [35,36]. A subset of these proteins physically interacts with Vgsc in mammals [28]. Five ankyrin domain-containing proteins were also identified in our high association gene set (S2 Table). Finally, *NEDD4*, also a high association gene, has predicted physical interactions with N_AV_ (Vgsc) in mammals (S2 Table) [37]. Additional support for N_AV_-Nedd4 interactions was also observed in its regulation of cardiac N_AV_ [38]. *NEDD4* codes for E3 ubiquitin-protein ligase, which regulates degradation of plasma membrane proteins with PY motifs [39]. Aedine Vgsc has a partial PY motif (PPS, amino acids 1170–1172) rather than the canonical PY of mammalian N_AV_ proteins (PPSYDSV) [40]. Until now, no support for *VGSC-NEDD4* interactions has been reported for dipterans.

The importance of each of the associations summarized above was further substantiated by GSEA of each corresponding functional category. We tested each functional group for relative enrichment within the high association set compared to all coding genes. This was done using a hypergeometric test, which models a binomial distribution and tests the probability of a gene being represented more often than expected by chance [41]. Specific results are described below in the context of each functional group.

#### Selected gene silencing and phenotype validation

**Fig 4:**
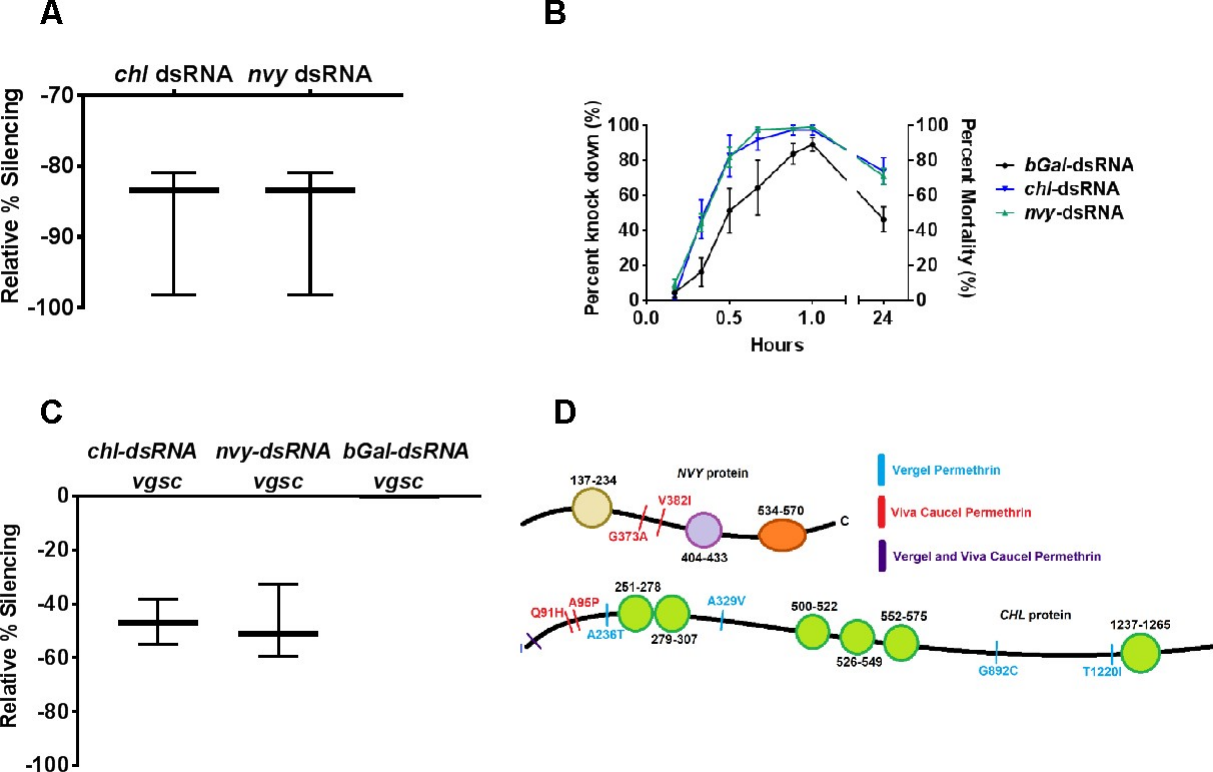
*CHL-* and *NVY*-silenced mosquitoes show increased susceptibility to permethrin and reduced VGSC transcript levels. A) Gene silencing validation. Mosquitoes were injected with either *chl-*dsRNA or *nvy-*dsRNA and processed in pools (n = 5) to isolate RNA at 3 days-post-treatment (dpt). Target transcripts were silenced an average of 93% and 78% in *chl*-dsRNA and *nvy*-dsRNA mosquitoes, respectively, relative to *βGal*-dsRNA-injected controls. B) Mosquitoes were injected with *βGal*-dsRNA (n = 113), *chl*-dsRNA (n = 172) or *nvy*-dsRNA (n = 129). At 3 dpt, each replicate was subjected to ~1.5 ug permethrin in a CDC bottle assay; knockdown was recorded at 10 minute intervals. There was a significant difference between *chl* and *nvy* compared to *β-Gal* controls ***, (ANOVA, *CHL*, *p*<0.0001; *NVY*, *p*<0.0001). At 24 hours, mortality was also significantly elevated (Fisher’s Exact Test, *CHL*, *p*<0.0001, *NVY*, *p* = 0.001). Left Y axis indicates percent knockdown from 10–60 minutes. Right Y-axis indicates percent mortality at 24 hours. Error bars indicate standard error of the mean (SEM). Bottle assay data represent a compilation of 4 to 5 biological replicates. C) Pools of 5 *chl*-dsRNA and *nvy*-dsRNA treated mosquitoes were subjected qRT-PCR of *VGSC* relative to *βGal*-dsRNA injected controls. Results shown are the average of 3 biological and 3 technical replicates.

To further explore the role of selected genes in pyrethroid resistance, we evaluated 2 putative transcription regulators in the high association data set. We hypothesized that when silenced, relevant components would result in increased pyrethroid susceptibility. One gene, *NVY* (LOC5563881), codes for a predicted central and peripheral nervous system-specific transcriptional regulator [42] with dual functions in axon guidance [43] and transcriptional repression [44]. The other gene, *CHL*, described above, is genetically associated with *VGSC* in drosophilids. We used RNA interference-induced gene silencing via dsRNA-injection to temporarily silence each gene in Vergel colony mosquitoes (F_24_). Silencing was confirmed relative to levels in non-specific dsRNA (*β-Gal-*dsRNA) treated controls (Fig 4A), and mosquitoes were subjected to a 1.5 μg permethrin discriminating dose in standard CDC bottle assays [45]. Both *nvy*-dsRNA and *chl*-dsRNA injected groups were significantly more susceptible to permethrin knockdown than (*β-GAL*- dsRNA)-injected controls (analysis of variance (ANOVA), *p*<0.0001) (Fig 4B). In contrast, PBS-injected controls showed sensitivity and viability levels that paralleled that of the *β-GAL*-injected group, indicating that exogenous dsRNA treatment alone had no effect (S1 Fig). The proportion of mosquitoes with lethal phenotypes at 24 hours post-treatment was also significantly greater in *nvy*-dsRNA and *chl*-dsRNA groups than that of *β-GAL-*dsRNA-injected mosquitoes (Fisher's exact test, *p* = 0.001 and *p*<0.0001, respectively) (Fig 4B).

Analysis of *VGSC* transcript levels showed a ~50% reduction following *nvy*-dsRNA or *chl*-dsRNA treatment compared to *β-GAL-*dsRNA-injected controls, indicating that each transcriptional regulator plays a role in modulating *VGSC* transcript levels (Fig 4C). The association mapping data was interrogated to identify *CHL* and *NVY* non-synonymous mutations in each of the collections. No non-synonymous mutations (LOD >4.0) were identified in either *CHL* or *NVY* in the VCD collection, suggestive of cis-regulatory effects. A single common mutation at A2T occurred in VCP and VeP, with a few other isolated amino acid substitutions in *CHL* (Fig 4D).

#### Detoxification and metabolic genes with high association to pyrethroid resistance

Detoxification is a common pyrethroid resistance mechanism [14,15,46–48], and several key enzymes in this category are within the metabolic functional group. Overall, seventy-eight high association genes had predicted activity in metabolic processes, as shown in Fig 2B (S2 Table), (78/1053), hypergeometric test *p* value = 0.02). Though type I, or non-cyano group bearing pyrethroids, eg., permethrin, and type II, or alpha-cyano group bearing, eg., deltamethrin, have distinct toxicity mechanisms [30], individual detoxification genes may respond to both types I and II [49]. The major pyrethroid detoxification proteins are mixed function oxidases and alpha- and beta-esterases (S2 Table) [50,51].

In anophelines, an acetylcholine esterase resistant allele (*ACE-1*^*R*^) can partially reduce deltamethrin sensitivity, and ace-1^R^ works synergistically with *VGSC* resistant alleles to substantially reduce sensitivity to permethrin [52]. However, there is little data to indicate an association between selection of acetylcholine esterase alleles and permethrin resistance in *Ae*. *aegypti*. Nevertheless, two acetylcholine esterases were included in the high association group (S2 Table, LOC5570776 and LOC5578456). Three genes coding for carboxylesterases (COEs, LOC5564561, LOC5576941, LOC5571034) were also in this group. COEs have been tied to organophosphate resistance mechanisms and, to a lesser extent, to deltamethrin resistance [53]. In addition to supporting a role for these genes in pyrethroid detoxification, their presence in the common gene set is consistent with the idea that there may be some overlap between type I and II pyrethroid detoxification mechanisms. In contrast, there were no epilson class glutathione S transferases in the high association group, which is surprising, given that they have been shown to be pertinent to resistant phenotypes [12,14,15,54–56].

Over-representation of the metabolic gene category is consistent with studies in drosophilids wherein amino acid catabolism was tied to permethrin sensitivity [57]. Specifically, Brinzer et al. found a decrease in proline levels in resistant larvae that had been exposed to permethrin, which could be explained by either increased conversion from proline to glutamate or inhibition of the proline precursor ornithine. Conversion of proline to glutamate has also been reported upon exposure to malathion or DDT [58,59]. In the current work, the aedine ortholog of 5-oxoprolinase, responsible for the degradation of glutamate, which would be required if it was in excess, is present in the high association group (S2 Table) [57].

Additional metabolic pathway genes in the high association group included lipid biosynthetic genes (S2 Table), for which transcripts are reportedly enriched in temephos-resistant mosquitoes [60]. Interestingly, there were also multiple genes that participate in lipid metabolic processes, ie., 3 lipases and 4 phospholipases (S2 Table). Increased lipase activity has been tied to insecticide resistance in *Tribolium spp* [61]. One possible explanation of this is that the catabolism of choline phospholipids is protective against the loss of choline, caused by the release of acetylcholine from synaptosomes [62] and inhibition of acetyl cholinesterase [63] that occurs in pyrethroid-susceptible insects. Similarly, a sphingomyelin phosphodiesterase (SMAse) activator domain protein was also in the high association group. SMAses are hydrolases that break down sphingomyelin into phosphocholine and ceramide (reviewed in [64]).

Some pyrethroid detoxification proteins are in the redox functional category. For our analyses, the redox and mitochondrial-associated gene subsets were combined, as key redox genes are localized to mitochondria. This category was not over-represented compared to all coding genes. Nevertheless, genes within this high association group included eighteen confirmed or putative *CYPs*, eight of which are *CYP4c* orthologs. *CYP4c* is an insect-specific member of the *CYP4* family; *CYP4* family proteins are best known for oxidation of fatty acids and localize to peroxisomes and the endoplasmic reticulum. Recent work has also reported their involvement in permethrin resistance [16]. Though *CYPs* do not directly detoxify permethrin, they may function downstream of esterases, which are responsible for much of permethrin catabolism (reviewed in [50]). Moreover, *CYP6* and *CYP9* (S2 Table) are also well documented for their involvement in resistance to both deltamethrin and permethrin [48,49].

Though *CYPs* are the best studied of the redox functional group [10,11,13,15], mitochondrial function genes also have been implicated in permethrin resistant anopheline mosquitoes, as indicated by the presence of elevated reactive oxygen species [46]. In addition, key electron transport gene cytochrome B (*CYTB*) transcripts are elevated in *Ae*.*aegypti* following permethrin treatment [65], consistent with a requirement for increased mitochondrial function. Here, there were five genes coding for cytochrome B and C associated genes in the high association subset (S2 Table).

#### Transport genes with high association to resistance

The transport functional category was the most statistically significant of all over-represented categories evaluated (120/1053, hypergeometric test *p* value = 1.3 x 10^−6^). This is intriguing, because one would not necessarily expect the category to be over-represented among all coding genes. Because the major pyrethroid target site, Vgsc, is a sodium transporter [5,66–71], it is possible that off-target receptor/channel functions are perturbed upon pyrethroid exposure and thus selected. Indeed, 55 of 120 genes in this category code for transporter, receptor or channel proteins. An alternate possibility is that selection of transport genes represents compensatory mutations to restore overall membrane homeostasis disrupted by pyrethroid binding to Vgsc in polarized neuronal cells (reviewed in [72]).

#### Repair/replication/transcription/translation genes

The largest specific functional category was for genes that affect DNA replication/ repair/transcription/translation (RRTT) functions. About 12.7% of all common genes were in this category, and though they were not over-represented relative to all coding genes (134/1053), there was a trend toward enrichment (hypergeometric test *p* value = 0.066). Of these genes, twenty are predicted to regulate transcription, including *CHL*, described above. Two orthologs of the transcription factor *grauzone* were also in this group. Grauzone is a Cys2His2 zinc-finger positive regulator of transcription and is required for meiosis in oogenesis [73]. Its possible role in insecticide resistance is unknown.

The effects of changes in transcriptional regulation of pyrethroid resistance are understudied in mosquitoes. Within the RRTT functional group, ribosomal protein subunits, histone proteins and histone methyltransferases were identified, hinting that insecticide resistance may require alterations from chromatin modifications to the initiation of gene expression to translational processes for establishment of the resistant phenotype.

#### Cytoskeletal/structural genes

Cytoskeletal components are crucial to proper neuronal function (reviewed in [74]). Genes coding for predicted cytoskeletal/structural function proteins were not over-represented among the subset common to all three collections. However, specific cytoskeletal/structural category genes with ankyrin domains are predicted to interact with Vgsc [27,28,33] and are genetically associated with *PARA*^*bss*^ in *Drosophila spp*. [27]. In addition, *sickie* (LOC5564933), a cytoskeletal positive regulator of neuronal axon growth, is also in the high association set [75], as was β-spectrin (*β-SPEC*) (S2 Table), a cytoskeletal protein important to maintaining neuronal structure in mammals and dipterans [74,76]. In mammals, *β-SPEC* is a neuronal structural component that physically interacts with N_AV_ channels [33] and also affects presynaptic stability [33].

#### Signaling

Genes with predicted function in cellular signaling were significantly over-represented (31/1053, hypergeometric probability *p* value = 0.013). G-protein coupled receptors (GPCR) are suspected of activating expression of *CYPs* in permethrin resistant *Culex quinquefasciatus* [77]. Twelve of 24 genes in this subset were among the high association set; these included GPCRs, GTP-binding proteins and GTPases. Second messenger signaling components are also important to overall control of cytoskeletal changes required for neural synapse function (reviewed in [74]).

#### Genes with exceptionally high association levels

To identify the highest resistance-associated genes among all treatment groups (VCP, VCD and VeP), genes among the top 10% of–log_10_(*p* value) values in the high association set were identified. Values within this extreme group were all greater than 9.02, 7.95 and 9.85 for VCP, VCD and VeP, respectively. Thirteen genes, including *VGSC*, were within this subset, and eight of these were located on the *p* arm of chromosome 3, in the same region as *VGSC*. Therefore, extreme association on the *3p* arm may be due to a selective sweep of *VGSC*, resulting in incidental selection of genes that have no functional association. However, if this was the case, we would expect a higher percentage of the 737 high association genes located on the chromosome *3p* arm to be included in the extreme group. Specifically, the extreme association group represents just 1.7% of the *VGSC* -proximal high association cluster. This is consistent with the hypothesis that key genes within this subset are specifically selected due to functional attributes rather than by proximity to *VGSC* alone. Importantly, these observations do not negate the possibility of a selective sweep in this region.

In addition to *VGSC*, the extreme association group included two transcriptional regulators (*NERVY* (*NVY*) LOC5563881 and LOC5579659), a Gr14 gustatory receptor (LOC5575007), a putative lipid binding protein, neural *LAZARILLO* (LOC5572156), *CINGULIN* (LOC5577979), a structural component of gap junctions at neuronal synapses [78] and three hypothetical genes. *LAZARILLO*, a lipid-binding apolipoprotein, though not studied in mosquitoes, is a biomarker for deltamethrin liver toxicity in mammals [79]. For all of these gene products, the mechanisms of association with pyrethroid resistance in mosquitoes remain to be explored.

#### Genes associated with synaptic function

Permethrin binding to the neuronal-specific transmembrane protein, Vgsc, could result in synaptic injury. The resulting toxic effects may select for mutations in genes that control pre- and post- synaptic processes. To explore this hypothesis, the high association group was interrogated for the presence of genes with known association to pre- and post-synaptic function. Twenty-one genes with demonstrated associations to this process were identified (S2 Table) [74,80,81]. Calcium signaling is requisite to motor neuron function [82,83], and the characteristic phenotype of pyrethroid treatment is the loss of motor control, due to insecticide binding to Vgsc. Therefore, the genetic association of both *CAa1* and *VGSC* to pyrethroid resistance is consistent with complementary contributions of each protein toward motor synaptic function. In the drosophilid model, *PARA* expression is stimulated upon limiting synaptic activity levels [83]. Consistent with these observations, the translational repressor, *PUM*, helps regulate motoneuronal activity, a function that is conserved in mammals and dipterans [84,85]. It does this via control of *VGSC* (*PARA*) translation, thus regulating synaptic excitation [83]. Of note, *PUM* was also present in our high association set, consistent with a role in the selection of resistance (S2 Table). Synaptotagmins were another gene cluster associated with synaptic function; these genes code for synaptic vesicle calcium sensors (S2 Table) [86] that interact with N_AV_ in mammals [87].

Neuronal cytoskeletal genes are crucial to maintenance of synaptic control (reviewed in [74]). Several cytoskeletal and associated regulatory genes important to synaptic activity were in the high association set (S2 Table), including an atypical protein kinase C (aPKC) ortholog (LOC5576493). aPKC stabilizes the microtubule cytoskeleton for proper synaptic function (reviewed in [74]). *β-SPEC* is an important structural feature of synaptic connections [35] and interacts with N_AV_ channels in mammals [88]. Moreover, neuroligin-1 was also present in the high association group, also consistent with selection at excitatory synapses [89].

## Conclusions

**Fig 5:**
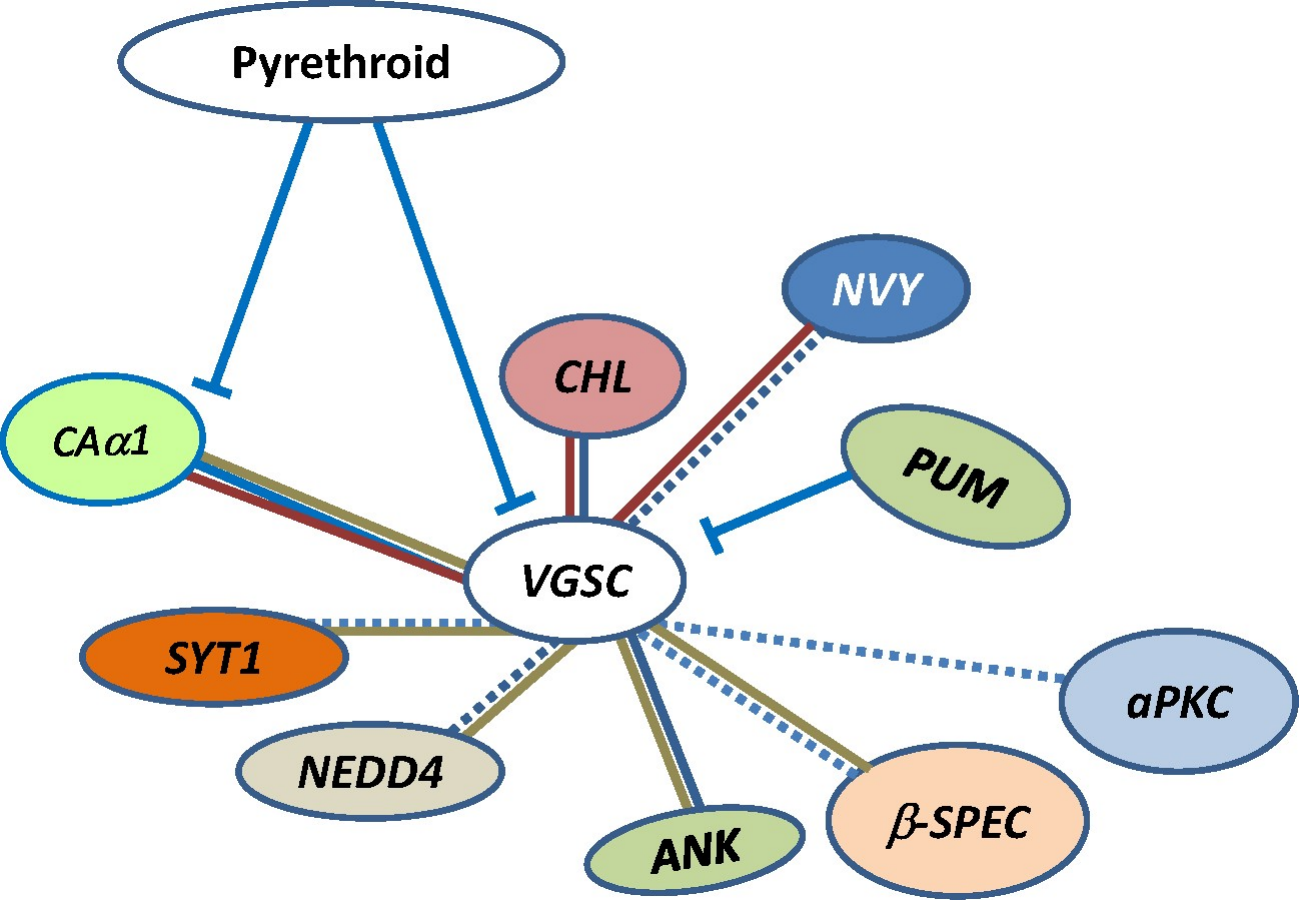
Putative interacting genes were inferred from 1) their presence in the high association set, and 2) functional or genetic evidence in model organisms or 3) reported here for *NVY* and *CHL* in *Ae*. *aegypti*. *ANK*, ankyrin-2 [33,35]; *aPKC*, atypical Protein kinase C [74]; *β-SPEC*, beta-spectrin [35,74,76]; *CAa1*, calcium channel alpha subunit [30,31]; *CHL*, *CHARLATAN* [25,26]*; NEDD4*, E3 ubiquitin-protein ligase [37,38]; *NVY*, *NERVY* [42–44], *PUM*, *pumilio* [27], *SYT1*, *SYNAPTOTAGMIN-1* [87,90]; *VGSC*, voltage-gated sodium channel [74]. Dotted line indicates indirect interaction due to shared roles in synaptic function. Blue line indicates genetic association in drosophilids. Red line indicates genetic evidence in *Ae*.*aegypti* or mosquito cell culture. Brown line indicates evidence in mammals.

We identified gene subsets with significant enrichment in populations resistant to pyrethroids. Importantly, genes were also identified that genetically, functionally or physically interact with Vgsc in other model organisms or in cell culture. Genes which were present in the high association set and for which multiple lines of evidence support genetic or physical interactions with *VGSC* are highlighted in Fig 5 [25,26,28,32,33,37]. We also found that aedine transcriptional regulators *CHL* and *NVY* are associated with permethrin resistant phenotypes. This is consistent with the hypothesis that transcriptional processes are under selective pressure. Both *CHL* and *NVY* are located on the *p* arm of chromosome 3 (red arrows, Fig 3) near *VGSC* within the area of a proposed selective sweep. The functional associations described in this report are consistent with the idea that specific genes within the *VGSC*-proximal cluster are associated with resistant phenotypes, are under selective pressure, and are not selected merely due to chromosomal proximity.

One explanation for over-representation of the “Transport” functional group is that polarized neuronal cells are more sensitive to alteration of transport functions and therefore under selective pressure in pyrethroid treatment areas. Mosquitoes may develop compensatory mutations in genes coding for transport proteins in an effort to reduce cell toxicity caused by synaptic damage. Though not studied in dipterans, patch clamp analyses of rat Na_v_1.8 sodium channel isoform in *Xenopus* oocytes indicated that membrane depolarization was not significantly impacted by permethrin or deltamethrin treatment, however there were significant decreases in calcium influx and glutamate release that were similar for both permethrin and deltamethrin [30]. Thus, taken together with the evidence above, we hypothesize that, in addition to *VGSC*, channels/transporters involved in glutamate and calcium homeostasis are major drivers of selection of transport category genes that contribute to resistance.

The genetic associations identified here need to be more comprehensively validated in mosquitoes to further support their roles in pyrethroid resistance.

## Materials and methods

### Collections and bioassays

Both populations used for this work were from the Merida region of Yucatan, Mexico and were collected from locations that were 13 kilometers apart. Population #1 was from Viva Caucel (20°59'57.83"N 89°42'15.41"W) was collected in 2011 by María Alba Loroño-Pino and Julian García-Rejón of Universidad Autónoma de Yucatán. The original larval collection (n = 60) was obtained from ~20 containers; more details are reported in Saavedrea-Rodriguez et al. 2015 [7]. Progeny from this collection was reared two generations in the laboratory. The third generation (about 3000 eggs) was reared to adulthood; a subset (Table 1) was exposed to permethrin-coated bottles using an amount that approximated the LC_50_ (lethal concentration, 50%, 25 μg per bottle active ingredient, Chem Service, West Chester, PA) [8]. Bioassays consisted of treatment of ~50 female mosquitoes (3–4 days old) for one hour [45]. Active mosquitoes, which were not knocked down (resistant), were transferred to cardboard cups and frozen at -80ᵒC. Mosquitoes that were knocked down were transferred to a second cardboard cup and placed into an incubator at 28ᵒC and 70% humidity. After a four hour recovery period, mosquitoes that remained inactive were scored as susceptible. DNA was isolated from individual mosquitoes using a salt extraction method [91] and resuspended in 150 μl of TE buffer (10 mM Tris-HCl, 1 mM EDTA pH 8.0). We typically isolate DNA from individual mosquitoes for archival purposes. For the current work, individual mosquito DNAs were carefully measured to allow for equimolar amounts of each mosquito in the sequencing libraries (see below).

Population #2 was also from Viva Caucel and was treated in bottles coated with 3 μg deltamethrin, also the LC_50_, using the same scheme as described above. Population #3 was from Vergel, Yucatán State in Southern Mexico (20°57'30.08"N, 89°35'4.33"W). It was also collected in 2011 by Universidad Autónoma de Yucatán and had been through two generations in the laboratory. The original larval collection (n = 47) was sampled from ~20 container sites [7]. Progeny (about 3000 eggs) from the 3^rd^ generation of this collection was reared to adulthood; a subset (Table 1) were treated with permethrin, using the same conditions, as described above for population #1.

### Gene silencing/bioassays

A 500 base-pair region of *CHL* or *NVY* was synthesized and inserted into a bacterial plasmid, pUC-IDT_kan_(Integrated DNA technologies). dsRNAs were generated using T7 primers appended to gene-specific primers, using the methods of Campbell et al. [92], wherein T7 dsDNA was prepared from synthesized gene fragments, and dsRNA was subsequently transcribed from the dsDNA. All primers are listed in S3 Table. Three to seven day old Vergel colony mosquitoes (F_24_) were injected with ~400 ng dsRNA and assayed at 3 days post-injection. Gene silencing was validated by qRT-PCR. Briefly, total RNA was extracted from pools of 5 mosquitoes (Qiagen RNAEasy kit), and 10 ng RNA was used as input into each 20 μl qRT-PCR reaction (NovaScript, Qiagen). Data was compiled from at least three biological replicates and three technical replicates for each treatment group. Average values for amplified ribosomal protein S7 (RPS7) and *aaeACTIN* were used for normalizing quantitation of relative expression using the delta-delta Ct relative gene expression calculation, relative to *β-Gal-*dsRNA injected control mosquitoes. At 3 days-post-injection, pools of mosquitoes were subjected to standard CDC bottle assays [45] using the discriminating dose of 1.5 μg permethrin per bottle (Sigma-Aldrich). Because the colony had been maintained for 24 generations in the lab without permethrin selection, the population had regained substantial sensitivity to insecticide treatment. Knockdown was measured in 10 minute increments for one hour, and mortality was scored at 24 hours post-treatment. Knockdown data represent a compilation of 5 experimental replicates.

### Pooling and quantification of samples

For each location/treatment group, gDNA from 25 resistant females was pooled for each of two replicates; similarly gDNA from 2 pools of 25 susceptible females was prepared. Before pooling, DNA from each individual mosquito was quantified using the Quant-IT Pico Green kit (Life Technologies) and ~40 ng from each individual DNA sample (25 individuals per library) was used for a final DNA pool of 1 μg. A Covaris S2 sonicator (Covaris Ltd, Brighton UK) sheared pooled DNA to an average size of 500 bp. Sonication conditions were: duty cycle 10%, Intensity 5.0, Cycles per burst 200, Duration 40 seconds, Mode Frequency sweeping, Displayed Power 23W, Temperature 5.5° - 6°C. Each TruSeq DNA LT (v.2) library was prepared using 1 μg of sheared genomic DNA following manufacturer’s recommendations. We prepared one library for each of the twelve DNA pools following the Illumina TruSeq DNA Sample preparation guide (Illumina, San Diego CA).

Equimolar quantities of prepared libraries were pooled and enriched for coding sequences by exome capture using custom SeqCap EZ Developer probes (Nimblegen) [19]. In total, 26.7Mb of the genome (2%) was targeted for enrichment, as described elsewhere [20]. Overlapping probes covering the protein coding sequence (not including UTRs) in the AaegL1.3 gene annotations (https://www.vectorbase.org/organisms/aedes-aegypti/liverpool-lvp/AaegL1.3) were produced by Nimblegen. Enrichment followed the Nimblegen SeqCap EZ protocol. Briefly, pooled TruSeq libraries were hybridized to the probes for 64 hours at 47°C, unbound DNA was washed away, and the targeted DNA was eluted and amplified. These were then sequenced on 2 lanes of a HiSeq2500 (Illumina) for paired-end 2 x 100 nt sequencing by the Centers for Disease Control and Prevention and Control in Atlanta, GA, producing reads with quality scores > 30.

### Bioinformatics

#### Alignments and population genetics pipeline

The analytical methods reported here have been used previously to identify polymorphic differences between aedine subspecies and sex-specific polymorphisms [19,93]. Though the published versions of the papers reported F_ST_ values rather than contingency χ^2^ –log_10_(p values), preliminary analyses of–log_10_(p values) showed trends identical to those in the published reports. Importantly, the sex-specific polymorphisms we described were also replicated independently by others [94]. All raw reads were trimmed of adapters and filtered using cutadapt [95]. The AaegL5 genome build [21] of 18,081 coding and non-coding genes was used, including all 5’UTRs, exons, introns, 3’UTRs. The 5’ and 3’ non-transcribed regions in previously reported alignments were excluded [19]. Individual replicate trimmed fastq files were aligned to a custom reference file using GSNAP (version 2013-10-28), allowing 10% divergence [96]. GSNAP outputs were converted to *.mpileup files, using SAMtools [97]. The “readcounts” command in Varscan2 (v2.3.5) [98] was used to convert *.mpileup files to readcounts output, using the following options:—min-coverage 15—min-base-qual 30. FORTRAN programs used in this analysis are available upon request. The number of aligned reads was determined in .bam files, using SAMtools flagstat and multiplied by the read-length (100 nt) to achieve the total nucleotides aligned. The ratio of variant sites per chromosome was multiplied by 1000 and divided by the total aligned nucleotides (S1 Table) to obtain the “Ratio sites/aligned nucleotide * 1000.”

Because the probes were designed using genome build L1.3, and the current reference genome is L5, we checked one of the libraries (VCDA-rep1) to determine the percentage and depth of sequencing for capture target sequences that aligned to L5. Coverage depth files of .bam files were generated using SAMtools, then the analogous L5 capture coordinate intervals were located. About 15.3% (chr 1), 6.5% (chr 2), and 11.7% (chr 3) of L5 capture targets were not either not sequenced or had coverage less than the 15 read coverage cut-off. In addition, a blastn search of L5 transcripts using the capture probes indicated that they cover (13,942/18,081) 77% of the genes in L5.

All sequencing data generated under this project are available at the National Center for Biotechnology Information (NCBI) Sequence Read Archive, Bioproject accession number PRJNA393171. VCPA permethrin-treated resistant replicates 1 and 2 are SRR5805471 and SRR5805472, respectively. VCPD permethrin-treated susceptible replicates 1 and 2 are SRR5805473 and SRR5805470, respectively. VCDA deltamethrin-treated resistant replicates 1 and 2 are SRR5805467 and SRR5805466, respectively. VCDD deltamethrin-treated susceptible replicates 1 and 2 are SRR5805469 and SRR5805468, respectively. VePA permethrin-treated resistant replicates 1 and 2 are SRR5805465 and SRR5805464, respectively. VePD permethrin-treated susceptible replicates 1 and 2 are SRR5805475 and SRR5805474, respectively.

In-house FORTRAN scripts were used to calculate heterogeneity χ^2^ values with degrees of freedom equal to the number of alternate nucleotides minus 1. The contingency χ^2^ calculation tested for the presence of an alternate nucleotide at each position along the genome and computed a -log_10_(*p* value) at each SNP [19]. For the purpose of highlighting genes with high statistical association to pyrethroid resistance, calculations of weighted gene-wise average scores were made by determining the mean of the top 5% -log_10_(*p* value) values per gene. Our rationale for this calculation was as follows. Along the length of a given coding sequence, many nucleotide sites showed little change relative to the reference sequence, thus resulting in many -log_10_(*p* value)at or near zero for a given SNP. Coding changes indicative of selection toward pyrethroid resistance may be present in a small subset of sites relative to the full length gene. Therefore, a weighted average allowed us to identify those genes with localized high value SNPs. Statistical significance was determined using the Benjamini-Hochberg False Discovery Rate [22] (FDR, alpha = 0.01) and a cut-off of 4.0 was established for gene-wise averages.

Gene annotations were obtained from Vectorbase [23] using BioMart. Functional groups were assigned to the following categories: ‘apoptosis’(APOP); ‘chemosensory response‘ (CSR); cytoskeletal/structural (CYT/STR); ‘diverse’ (DIV) for genes with multiple or less clearly defined function(s); ‘mitochondrial’ (MIT) for gene products localized to mitochondrial compartment, regardless of function; (LIPID) for genes with predicted function in lipid processing or biosynthesis; (ReDox) for genes with predicted function in oxidation/reduction processes; proteolysis or proteosomal activity (PROT); replication/(DNA)repair/transcription /translation’ (RRTT); signal transduction (SigT); metabolism (MET); ‘unknown’ (UNK) for uncharacterized genes. Finally, the ‘transport’ (TRP) category included all gene products predicted to be involved in moving molecules across membranes, including receptors, exclusive of secondary messengers and signaling receptors. For GSEA, the mitochondrial and redox categories were combined, as several key redox activities occur in proteins localized to mitochondria.

#### Statistics

Descriptive statistics were calculated in R (version 3.0.2). The ratio of variant sites per nucleotide of aligned reads was calculated as follows: binomial probability distributions were performed and hypergeometric tests were calculated; each functional subset was compared to the corresponding group within the set of all coding genes (n = 14,626). This analysis allowed us to identify the gene sets that were enriched compared to similar categories among all coding genes.

## Supporting information

S1 Table Sequencing details for all libraries. Number of reads aligned to reference, percent trimmed reads mapped to reference, number of variant sites per chromosome, ratio of sites per aligned nucleotide * 1000.
(XLSX)

S2 Table χ^2^ contingency values for all collections in the high association set (n = 1053). Genes are arranged in order of their physical position. Genes in the top ten percent of all common genes are highlighted in gray, those with predicted motoneuron synaptic function are highlighted in pink, and predicted transcriptional regulators are highlighted in green. RefSeq locus, chromosome, function, aliases, description, genomic_nucleotide_accession, start_position, end_position, # exons. For each collection- “_pvalue”, weighted average–log(p value) per gene; AvgHexp_Resistant; AvgHexp_Susceptible.
(XLSX)

S3 Table Primers used in gene silencing and qRT-PCR. (XLSX)

S1 Fig *CHL-* and *NVY*-silenced mosquitoes show increased susceptibility to permethrin. Mosquitoes were injected with phosphate-buffered saline (PBS, n = 109), *βGal*-dsRNA (n = 113), *chl*-dsRNA (n = 172), *nvy*-dsRNA (n = 129) or left untreated (n = 136). At 3 dpt, each replicate was subjected to ~1.5 ug permethrin in a CDC bottle assay; knockdown was recorded at 10 minute intervals. Left Y axis indicates percent knockdown from 10–60 minutes. Right Y-axis indicates percent mortality at 24 hours. Error bars indicate SEM. Data represent a compilation of 4 to 5 biological replicates.
(TIF)
