# Supplementary figures and images for: Vgsc-interacting proteins are genetically associated with pyrethroid resistance in Aedes aegypti
Source: PLoS One. 2019 Jan 29;14(1):e0211497. doi: 10.1371/journal.pone.0211497 (PMC6350986; doi:10.1371/journal.pone.0211497)

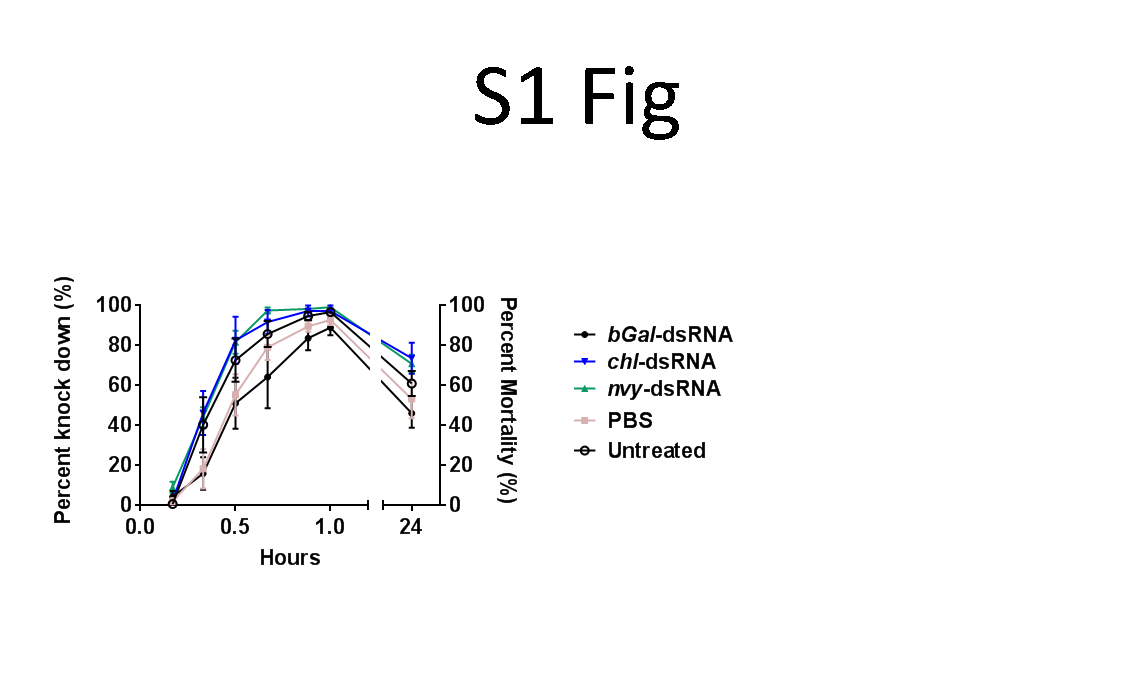

Supplement: S1 Fig — Mosquitoes were injected with phosphate-buffered saline (PBS, n = 109), βGal-dsRNA (n = 113), chl-dsRNA (n = 172), nvy-dsRNA (n = 129) or left untreated (n = 136). At 3 dpt, each replicate was subjected to ~1.5 ug permethrin in a CDC bottle assay; knockdown was recorded at 10 minute intervals. Left Y axis indicates percent knockdown from 10–60 minutes. Right Y-axis indicates percent mortality at 24 hours. Error bars indicate SEM. Data represent a compilation of 4 to 5 biological replicates. (TIF) [file pone.0211497.s004.tif]
